# Supplementary material for: Introducing SoNHR–Reporting guidelines for Social Networks In Health Research
Source: PLoS One. 2023 Dec 14;18(12):e0285236. doi: 10.1371/journal.pone.0285236 (PMC10721040; doi:10.1371/journal.pone.0285236)
Supplement: S1 File — (DOCX) [file pone.0285236.s001.docx]

Appendix A. Delphi survey for feedback on initial set of network reporting guidelines.

Section I: SNA Reporting Guidelines Ratings

For each of the guidelines displayed below, we ask that you rate two aspects: *importance* and *clarity*.

- *Importance* reflects the degree to which you feel that the particular guideline is critical for researchers to understand and follow in their empirical studies which apply SNA methods.
- *Clarity* reflects the degree to which the wording of the guideline clearly communications to researchers using these reporting guidelines the type of information needed to satisfy the guideline.

## Conceptualization

|  | Importance | Clarity |
| --- | --- | --- |
|  |  |  |
| Clearly describe the study's guiding theories, frameworks, or models so that it is clear how and why networks are important for the study. | ▼ 1 - Not at all Important  2 - Slightly Important  3 - Moderately Important  4 - Important  5 - Very Important | ▼ 1 - Not at all Clear  2 - Slightly Clear  3 - Moderately Clear  4 - Clear  5 - Very Clear |
| Make the value of a network analysis apparent. What does a network analysis tell us that a more traditional approach would not capture? | ▼ 1 - Not at all Important  2 - Slightly Important  3 - Moderately Important  4 - Important  5 - Very Important | ▼ 1 - Not at all Clear  2 - Slightly Clear  3 - Moderately Clear  4 - Clear  5 - Very Clear |
| Describe the conceptual underpinnings of the study in a way that makes it clear what specific aspects of social networks (e.g., ties, network dynamics) are most relevant for addressing the study's research questions. | ▼ 1 - Not at all Important  2 - Slightly Important  3 - Moderately Important  4 - Important  5 - Very Important | ▼ 1 - Not at all Clear  2 - Slightly Clear  3 - Moderately Clear  4 - Clear  5 - Very Clear |

Operationalization

|  | Importance | Clarity |
| --- | --- | --- |
|  |  |  |
| Define the *nodes* to make it clear what a node represents. | ▼ 1 - Not at all Important  2 - Slightly Important  3 - Moderately Important  4 - Important  5 - Very Important | ▼ 1 - Not at all Clear  2 - Slightly Clear  3 - Moderately Clear  4 - Clear  5 - Very Clear |
| Define the *ties* so that it is clear what each type of tie represents. | ▼ 1 - Not at all Important  2 - Slightly Important  3 - Moderately Important  4 - Important  5 - Very Important | ▼ 1 - Not at all Clear  2 - Slightly Clear  3 - Moderately Clear  4 - Clear  5 - Very Clear |
| Define the boundaries of the network so that it is clear who is included and not included in the network. | ▼ 1 - Not at all Important  2 - Slightly Important  3 - Moderately Important  4 - Important  5 - Very Important | ▼ 1 - Not at all Clear  2 - Slightly Clear  3 - Moderately Clear  4 - Clear  5 - Very Clear |
| State clearly the basic type of network that is being analyzed (e.g., complete network, ego networks, affiliation/2-mode/bipartite networks). | ▼ 1 - Not at all Important  2 - Slightly Important  3 - Moderately Important  4 - Important  5 - Very Important | ▼ 1 - Not at all Clear  2 - Slightly Clear  3 - Moderately Clear  4 - Clear  5 - Very Clear |

Data Collection & Management

|  | Importance | Clarity |
| --- | --- | --- |
|  |  |  |
| Describe network data collection procedures and tools (e.g., surveys and software) in enough detail to replicate. When possible, provide access to all surveys, instruments, and tools used. | ▼ 1 - Not at all Important  2 - Slightly Important  3 - Moderately Important  4 - Important  5 - Very Important | ▼ 1 - Not at all Clear  2 - Slightly Clear  3 - Moderately Clear  4 - Clear  5 - Very Clear |
| Describe the network data used in the study, including pre-existing data sources, how the data are stored, managed, and whether/where they are publicly available. | ▼ 1 - Not at all Important  2 - Slightly Important  3 - Moderately Important  4 - Important  5 - Very Important | ▼ 1 - Not at all Clear  2 - Slightly Clear  3 - Moderately Clear  4 - Clear  5 - Very Clear |
| Discuss missingness in network data, its implications, and any attempts to impute or account for missing data. | ▼ 1 - Not at all Important  2 - Slightly Important  3 - Moderately Important  4 - Important  5 - Very Important | ▼ 1 - Not at all Clear  2 - Slightly Clear  3 - Moderately Clear  4 - Clear  5 - Very Clear |
| For organizational-level survey data where multiple responses represent an organization, describe any data aggregation or reduction methods. | ▼ 1 - Not at all Important  2 - Slightly Important  3 - Moderately Important  4 - Important  5 - Very Important | ▼ 1 - Not at all Clear  2 - Slightly Clear  3 - Moderately Clear  4 - Clear  5 - Very Clear |
| For valued networks, describe reconciliation of conflicting values when provided by both members of the dyad (e.g., min, max, mean). | ▼ 1 - Not at all Important  2 - Slightly Important  3 - Moderately Important  4 - Important  5 - Very Important | ▼ 1 - Not at all Clear  2 - Slightly Clear  3 - Moderately Clear  4 - Clear  5 - Very Clear |
| Describe the decision rule for cases when only one member of the dyad reports a relationship; provide rationale for requiring one or both responses. | ▼ 1 - Not at all Important  2 - Slightly Important  3 - Moderately Important  4 - Important  5 - Very Important | ▼ 1 - Not at all Clear  2 - Slightly Clear  3 - Moderately Clear  4 - Clear  5 - Very Clear |

## Analyses & Results

### Analyses & Results: Description

|  | Importance | Clarity |
| --- | --- | --- |
|  |  |  |
| Discuss the meaning of node-level statistics (e.g., degree, betweenness, closeness, etc.) or network-level statistics (e.g., centralization, degree distribution, component structure) in the context of the network, your structural/relational theories, or your research questions. | ▼ 1 - Not at all Important  2 - Slightly Important  3 - Moderately Important  4 - Important  5 - Very Important | ▼ 1 - Not at all Clear  2 - Slightly Clear  3 - Moderately Clear  4 - Clear  5 - Very Clear |
| When discussing network properties, explain the network statistics in enough detail so it is clear to the reader what specific measures were used and why they were chosen (e.g., which measure of homophily/modularity, weighted or unweighted degree for valued networks, etc.). | ▼ 1 - Not at all Important  2 - Slightly Important  3 - Moderately Important  4 - Important  5 - Very Important | ▼ 1 - Not at all Clear  2 - Slightly Clear  3 - Moderately Clear  4 - Clear  5 - Very Clear |

Analyses & Results: Visualization

|  | Importance | Clarity |
| --- | --- | --- |
|  |  |  |
| Use node and tie size, color, type, shape, and labels to focus attention on the most important structural aspects of the networks. | ▼ 1 - Not at all Important  2 - Slightly Important  3 - Moderately Important  4 - Important  5 - Very Important | ▼ 1 - Not at all Clear  2 - Slightly Clear  3 - Moderately Clear  4 - Clear  5 - Very Clear |
| Use network visualization best practices appropriate for the size of the network and the goal of the visualization (e.g., collapsing related nodes, limited use of labels and different shapes in large networks, and varying line weights or colors in small networks). | ▼ 1 - Not at all Important  2 - Slightly Important  3 - Moderately Important  4 - Important  5 - Very Important | ▼ 1 - Not at all Clear  2 - Slightly Clear  3 - Moderately Clear  4 - Clear  5 - Very Clear |
| Use node color or shape to convey categorical properties. | ▼ 1 - Not at all Important  2 - Slightly Important  3 - Moderately Important  4 - Important  5 - Very Important | ▼ 1 - Not at all Clear  2 - Slightly Clear  3 - Moderately Clear  4 - Clear  5 - Very Clear |
| Use node size to convey a quantitative property, either structural (e.g., centrality) or an actor attribute (e.g., income). | ▼ 1 - Not at all Important  2 - Slightly Important  3 - Moderately Important  4 - Important  5 - Very Important | ▼ 1 - Not at all Clear  2 - Slightly Clear  3 - Moderately Clear  4 - Clear  5 - Very Clear |
| Only label nodes if they are important for understanding the network; consider only labeling specific important nodes. | ▼ 1 - Not at all Important  2 - Slightly Important  3 - Moderately Important  4 - Important  5 - Very Important | ▼ 1 - Not at all Clear  2 - Slightly Clear  3 - Moderately Clear  4 - Clear  5 - Very Clear |

Analyses & Results: Modeling & Simulation

|  | Importance | Clarity |
| --- | --- | --- |
|  |  |  |
| If statistical network models are used, describe the model in enough detail so that readers can understand what the *outcome* of the model is (e.g., likelihood of observing a tie). | ▼ 1 - Not at all Important  2 - Slightly Important  3 - Moderately Important  4 - Important  5 - Very Important | ▼ 1 - Not at all Clear  2 - Slightly Clear  3 - Moderately Clear  4 - Clear  5 - Very Clear |
| Clearly distinguish between node-level, dyadic, and structural predictors (or *covariates*) in statistical network models. | ▼ 1 - Not at all Important  2 - Slightly Important  3 - Moderately Important  4 - Important  5 - Very Important | ▼ 1 - Not at all Clear  2 - Slightly Clear  3 - Moderately Clear  4 - Clear  5 - Very Clear |
| Present some information (possibly in appendices) on how well the network model fits with the observed network data (e.g., through simulations of fitted ERGM models, examination of MCMC diagnostics, etc.) and the implications of the model fit results you are reporting. | ▼ 1 - Not at all Important  2 - Slightly Important  3 - Moderately Important  4 - Important  5 - Very Important | ▼ 1 - Not at all Clear  2 - Slightly Clear  3 - Moderately Clear  4 - Clear  5 - Very Clear |
| For network simulations, describe the underlying mechanisms represented in the simulation and how these mechanisms relate to the relevant research questions. | ▼ 1 - Not at all Important  2 - Slightly Important  3 - Moderately Important  4 - Important  5 - Very Important | ▼ 1 - Not at all Clear  2 - Slightly Clear  3 - Moderately Clear  4 - Clear  5 - Very Clear |

Ethics & Equity

|  | Importance | Clarity |
| --- | --- | --- |
|  |  |  |
| Report and describe the informed consent process, including if and how respondents were offered informed consent. | ▼ 1 - Not at all Important  2 - Slightly Important  3 - Moderately Important  4 - Important  5 - Very Important | ▼ 1 - Not at all Clear  2 - Slightly Clear  3 - Moderately Clear  4 - Clear  5 - Very Clear |
| Discuss how confidentiality was explained to participants and how their confidentiality was ensured, including considerations of identifiability in network visualizations and reporting. | ▼ 1 - Not at all Important  2 - Slightly Important  3 - Moderately Important  4 - Important  5 - Very Important | ▼ 1 - Not at all Clear  2 - Slightly Clear  3 - Moderately Clear  4 - Clear  5 - Very Clear |
| When appropriate, discuss inclusion in the network study and results in terms of equity and social, economic, and health justice. | ▼ 1 - Not at all Important  2 - Slightly Important  3 - Moderately Important  4 - Important  5 - Very Important | ▼ 1 - Not at all Clear  2 - Slightly Clear  3 - Moderately Clear  4 - Clear  5 - Very Clear |
| Discuss any potential biases within network structures and results that may be rooted in data collection, (e.g., failure to capture complete networks, organizational or specific group non-participation, or over-representation). | ▼ 1 - Not at all Important  2 - Slightly Important  3 - Moderately Important  4 - Important  5 - Very Important | ▼ 1 - Not at all Clear  2 - Slightly Clear  3 - Moderately Clear  4 - Clear  5 - Very Clear |

Section II: SNA Reporting Guidelines General Feedback

Thank you for providing feedback on the specific reporting guidelines for each domain in Section I. We would also like your input on some broader considerations relating to this project, including issues you have observed as a producer and consumer of SNA empirical research literature (e.g., research papers, methodological papers and chapters, conference presentations, etc.), the overall utility of SNA reporting guidelines, and how best to promote and disseminate these guidelines to the field.

What problem(s) do you most frequently see in the presentation of network methods and results in health research?

________________________________________________________________

What is the single most important improvement you would like to see in the above draft SNA reporting guidelines?

________________________________________________________________

In what ways do you anticipate these reporting guidelines being helpful in your own work?

________________________________________________________________

What are your recommendations for disseminating these reporting guidelines to promote their use in the field?

________________________________________________________________

Is there anything else you would like to share with us about the SNA reporting guidelines?

_______________________________________________________________

Section III: Participant Information

Please answer the following questions about your professional and demographic background.

## Professional characteristics

In the past five years, have you done the following:

|  | Yes | No |
| --- | --- | --- |
| Published a paper which applies, describes, or extends SNA methods |  |  |
| Reviewed manuscripts or grant applications using SNA methods |  |  |
| Taught a class or workshop featuring SNA methods |  |  |
| Worked in an organization that funds health research using SNA methods (e.g. NIH) |  |  |
| Been a member of an SNA-focused professional organization (e.g., INSNA) |  |  |

How many years have you been using SNA methods in your research and evaluation activities?

- 1-5 years
- 6-10 years
- 11-20 years
- 20+ years

Which of the following options best describe the primary *disciplinary* focus areas for your research? [Check all that apply.]

- Anthropology
- Business
- Communications
- Economics
- Education
- Medicine
- Nursing
- Organizational research
- Political Science
- Psychology
- Public Health
- Public Policy/Administration
- Social Work
- Sociology
- Statistics, Mathematics, and/or Computer Science
- Other _____________________________

Which of the following options best describe the primary *health science applications* for your research? [Check all that apply.]

- Addiction, substance use
- Alternative medicines
- Brain, neuro sciences
- Cancer
- Cardiovascular disease
- Children, youth, and families
- Chronic disease
- Community-focused research
- Diabetes, obesity
- Disability
- Dissemination and implementation
- Epidemiology
- Global health
- Health disparities, equity, social justice
- Health policy
- Health promotion
- Health services
- Infectious disease
- Mental health
- Methods development
- Older adults
- Pharmacy
- Program evaluation
- Rural health
- Social services
- Tobacco cessation
- Urban planning
- Workforce development
- NA (Do not conduct health sciences research)
- Other _____________________________

Demographic characteristics

Please indicate your preferred gender identity:

- Man
- Woman
- Non-binary or third gender
- Self-describe: _________________
- Prefer not to say

Do you identify as transgender?

- Yes
- No
- Prefer not to say

What is your race? (Select one or more options)

- American Indian or Alaska Native
- Asian
- Black or African American
- Native Hawaiian or Other Pacific Islander
- White
- Additional race(s) not listed: _____________________
- Prefer not to say

Are you of Hispanic/Latinx ethnicity?

- Yes
- No
