# Supplementary material for: Introducing SoNHR–Reporting guidelines for Social Networks In Health Research
Source: PLoS One. 2023 Dec 14;18(12):e0285236. doi: 10.1371/journal.pone.0285236 (PMC10721040; doi:10.1371/journal.pone.0285236)

Appendix D. Technical details of rapid review of social network analysis in health literature.

The purpose of this rapid review was to provide context for the main goals of the SoNHR guideline development project. It would help us make the argument for focusing on the health sciences by demonstrating the rapid rise of network studies in health research.

Scopus was chosen as the database for the rapid literature review. Scopus is the world’s largest abstract and indexing database, and includes over 5,300 journals in the health sciences.

A quick Scopus search [“social network analysis” AND health] yields this for the last 20 years.

A more rigorous search string that includes specific health-related subject areas produces similar results:

TITLE-ABS-KEY ( "network analysis" )  AND  PUBYEAR  >  2001  AND  PUBYEAR  <  2022  AND  ( LIMIT-TO ( SUBJAREA ,  "MEDI" )  OR  LIMIT-TO ( SUBJAREA ,  "PHAR" )  OR  LIMIT-TO ( SUBJAREA ,  "HEAL" )  OR  LIMIT-TO ( SUBJAREA ,  "NURS" ) )


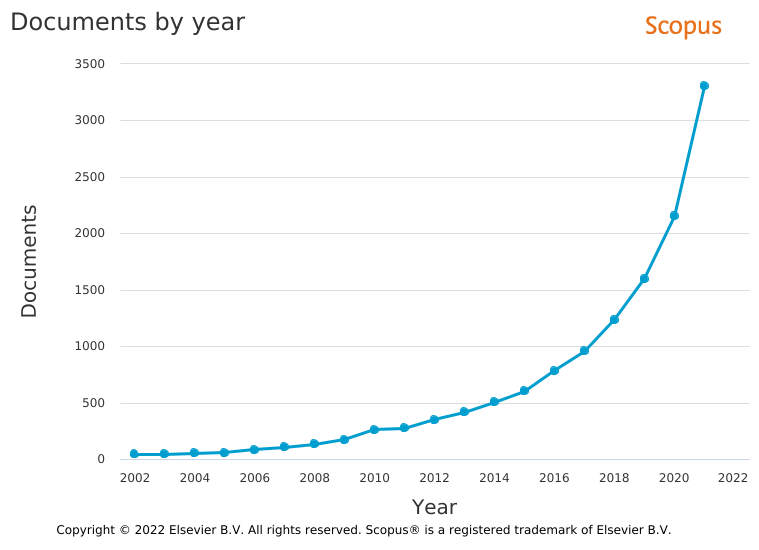


Not sure if we want to limit country at all?


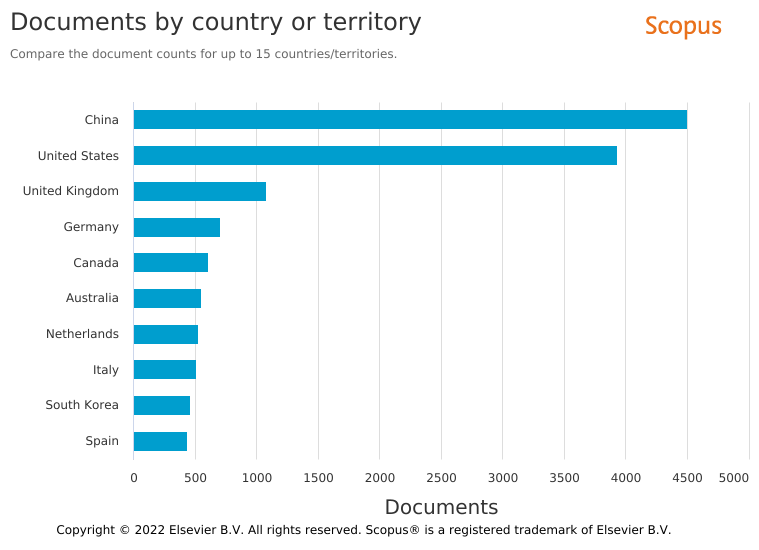


Documents can be multi-subject area, so we still get a few we don’t select:


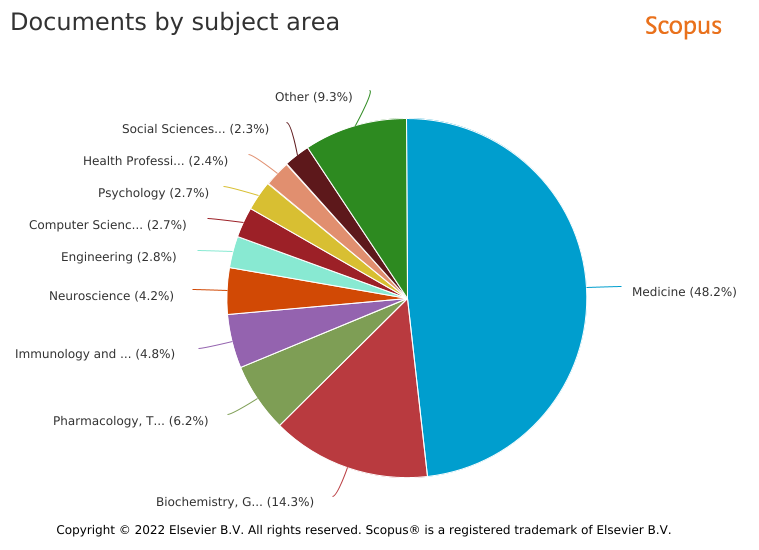


Combining previous searches gives us the ability to look at absolute and relative changes over time:

TITLE-ABS-KEY ( "network analysis" )  AND  PUBYEAR  >  2001  AND  PUBYEAR  <  2022

Network articles limited to health fields:

TITLE-ABS-KEY ( "network analysis" )  AND  PUBYEAR  >  2001  AND  PUBYEAR  <  2022  AND  ( LIMIT-TO ( SUBJAREA ,  "MEDI" )  OR  LIMIT-TO ( SUBJAREA ,  "PHAR" )  OR  LIMIT-TO ( SUBJAREA ,  "HEAL" )  OR  LIMIT-TO ( SUBJAREA ,  "NURS" ) )


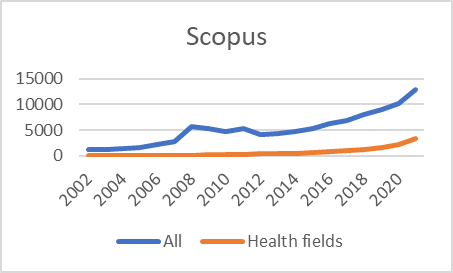

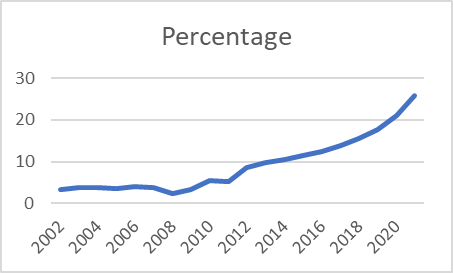

Supplement: S4 File — (DOCX) [file pone.0285236.s004.docx]
