# Supplementary material for: Introducing SoNHR–Reporting guidelines for Social Networks In Health Research
Source: PLoS One. 2023 Dec 14;18(12):e0285236. doi: 10.1371/journal.pone.0285236 (PMC10721040; doi:10.1371/journal.pone.0285236)
Supplement: S1 Table — (DOCX) [file pone.0285236.s005.docx]

Appendix B. Importance and clarity ratings for preliminary set of network reporting recommendations.

| Item | Scale | N | Mean | Median | SD |
| --- | --- | --- | --- | --- | --- |
| *Conceptualization* |  |  |  |  |  |
| 1. Clearly describe the study's guiding theories, frameworks, or models so that it is clear how and why networks are important for the study. | Importance | 67 | 4.57 | 5 | 0.61 |
|  | Clarity | 67 | 4.39 | 5 | 0.80 |
| 2. Make the value of a network analysis apparent. What does a network analysis tell us that a more traditional approach would not capture? | Importance | 67 | 4.10 | 4 | 1.06 |
|  | Clarity | 67 | 4.36 | 5 | 0.90 |
| 3. Describe the conceptual underpinnings of the study in a way that makes it clear what specific aspects of social networks (e.g., ties, network dynamics) are most relevant for addressing the study's research questions. | Importance | 67 | 4.15 | 4 | 0.84 |
|  | Clarity | 67 | 3.73 | 4 | 0.93 |
| *Operationalization* |  |  |  |  |  |
| 1. Define the nodes to make it clear what a node represents. | Importance | 67 | 4.90 | 5 | 0.31 |
|  | Clarity | 67 | 4.69 | 5 | 0.70 |
| 2. Define the ties so that it is clear what each type of tie represents. | Importance | 67 | 4.87 | 5 | 0.39 |
|  | Clarity | 67 | 4.67 | 5 | 0.70 |
| 3. Define the boundaries of the network so that it is clear who is included and not included in the network. | Importance | 67 | 4.72 | 5 | 0.67 |
|  | Clarity | 67 | 4.46 | 5 | 0.96 |
| 4. State clearly the basic type of network that is being analyzed (e.g., complete network, ego networks, affiliation/2-mode/bipartite networks). | Importance | 67 | 4.43 | 5 | 0.74 |
|  | Clarity | 67 | 4.39 | 5 | 0.92 |
| *Data Collection & Management* |  |  |  |  |  |
| 1. Describe network data collection procedures and tools (e.g., surveys and software) in enough detail to replicate. When possible, provide access to all surveys, instruments, and tools used. | Importance | 67 | 4.58 | 5 | 0.61 |
|  | Clarity | 67 | 4.51 | 5 | 0.79 |
| 2. Describe the network data used in the study, including pre-existing data sources, how the data are stored, managed, and whether/where they are publicly available. | Importance | 67 | 4.04 | 4 | 0.84 |
|  | Clarity | 67 | 4.15 | 4 | 0.91 |
| 3. Discuss missingness in network data, its implications, and any attempts to impute or account for missing data. | Importance | 67 | 4.45 | 5 | 0.63 |
|  | Clarity | 67 | 4.30 | 5 | 0.90 |
| 4. For organizational-level survey data where multiple responses represent an organization, describe any data aggregation or reduction methods. | Importance | 67 | 4.30 | 4 | 0.82 |
|  | Clarity | 67 | 3.85 | 4 | 1.26 |
| 5. For valued networks, describe reconciliation of conflicting values when provided by both members of the dyad (e.g., min, max, mean). | Importance | 66 | 4.14 | 4 | 0.88 |
|  | Clarity | 67 | 3.84 | 4 | 1.15 |
| 6. Describe the decision rule for cases when only one member of the dyad reports a relationship; provide rationale for requiring one or both responses. | Importance | 67 | 4.21 | 4 | 0.84 |
|  | Clarity | 67 | 4.04 | 4 | 1.01 |
| *Analysis & Results - Description* |  |  |  |  |  |
| 1. Discuss the meaning of node-level statistics (e.g., degree, betweenness, closeness, etc.) or network-level statistics (e.g., centralization, degree distribution, component structure) in the context of the network, your structural/relational theories, or your research questions. | Importance | 67 | 4.15 | 4 | 0.89 |
|  | Clarity | 67 | 4.03 | 4 | 0.89 |
| 2. When discussing network properties, explain the network statistics in enough detail so it is clear to the reader what specific measures were used and why they were chosen (e.g., which measure of homophily/modularity, weighted or unweighted degree for valued networks, etc.). | Importance | 67 | 4.37 | 5 | 0.79 |
|  | Clarity | 67 | 4.16 | 4 | 0.96 |
| *Analysis & Results - Visualization* |  |  |  |  |  |
| 1. Use node and tie size, color, type, shape, and labels to focus attention on the most important structural aspects of the networks. | Importance | 67 | 3.66 | 4 | 1.05 |
|  | Clarity | 67 | 4.22 | 4 | 0.88 |
| 2. Use network visualization best practices appropriate for the size of the network and the goal of the visualization (e.g., collapsing related nodes, limited use of labels and different shapes in large networks, and varying line weights or colors in small networks). | Importance | 67 | 3.78 | 4 | 0.90 |
|  | Clarity | 67 | 3.88 | 4 | 1.07 |
| 3. Use node color or shape to convey categorical properties. | Importance | 66 | 3.44 | 4 | 1.12 |
|  | Clarity | 66 | 4.32 | 5 | 0.93 |
| 4. Use node size to convey a quantitative property, either structural (e.g., centrality) or an actor attribute (e.g., income). | Importance | 66 | 3.38 | 4 | 1.11 |
|  | Clarity | 66 | 4.23 | 4 | 0.96 |
| 5. Only label nodes if they are important for understanding the network; consider only labeling specific important nodes. | Importance | 66 | 3.14 | 3 | 1.21 |
|  | Clarity | 66 | 3.97 | 4 | 1.12 |
| *Analysis & Results - Modeling & Simulation* |  |  |  |  |  |
| 1. If statistical network models are used, describe the model in enough detail so that readers can understand what the outcome of the model is (e.g., likelihood of observing a tie). | Importance | 67 | 4.70 | 5 | 0.49 |
|  | Clarity | 67 | 4.37 | 5 | 0.90 |
| 2. Clearly distinguish between node-level, dyadic, and structural predictors (or covariates) in statistical network models. | Importance | 67 | 4.48 | 5 | 0.64 |
|  | Clarity | 67 | 4.36 | 5 | 0.87 |
| 3. Present some information (possibly in appendices) on how well the network model fits with the observed network data (e.g., through simulations of fitted ERGM models, examination of MCMC diagnostics, etc.) and the implications of the model fit results you are reporting. | Importance | 66 | 4.17 | 4 | 0.87 |
|  | Clarity | 66 | 4.26 | 4 | 0.83 |
| 4. For network simulations, describe the underlying mechanisms represented in the simulation and how these mechanisms relate to the relevant research questions. | Importance | 65 | 4.29 | 4 | 0.82 |
|  | Clarity | 66 | 3.91 | 4 | 1.06 |
| *Ethics & Equity* |  |  |  |  |  |
| 1. Report and describe the informed consent process, including if and how respondents were offered informed consent. | Importance | 67 | 4.25 | 4 | 0.88 |
|  | Clarity | 67 | 4.54 | 5 | 0.88 |
| 2. Discuss how confidentiality was explained to participants and how their confidentiality was ensured, including considerations of identifiability in network visualizations and reporting. | Importance | 67 | 4.03 | 4 | 1.10 |
|  | Clarity | 67 | 4.40 | 5 | 0.85 |
| 3. When appropriate, discuss inclusion in the network study and results in terms of equity and social, economic, and health justice. | Importance | 67 | 3.82 | 4 | 1.04 |
|  | Clarity | 67 | 3.81 | 4 | 1.09 |
| 4. Discuss any potential biases within network structures and results that may be rooted in data collection, (e.g., failure to capture complete networks, organizational or specific group non-participation, or over-representation). | Importance | 67 | 4.60 | 5 | 0.58 |
|  | Clarity | 67 | 4.37 | 5 | 0.76 |
