# Supplementary material for: Introducing SoNHR–Reporting guidelines for Social Networks In Health Research
Source: PLoS One. 2023 Dec 14;18(12):e0285236. doi: 10.1371/journal.pone.0285236 (PMC10721040; doi:10.1371/journal.pone.0285236)

Appendix C. Distributions of importance and clarity ratings for preliminary set of network reporting recommendations.


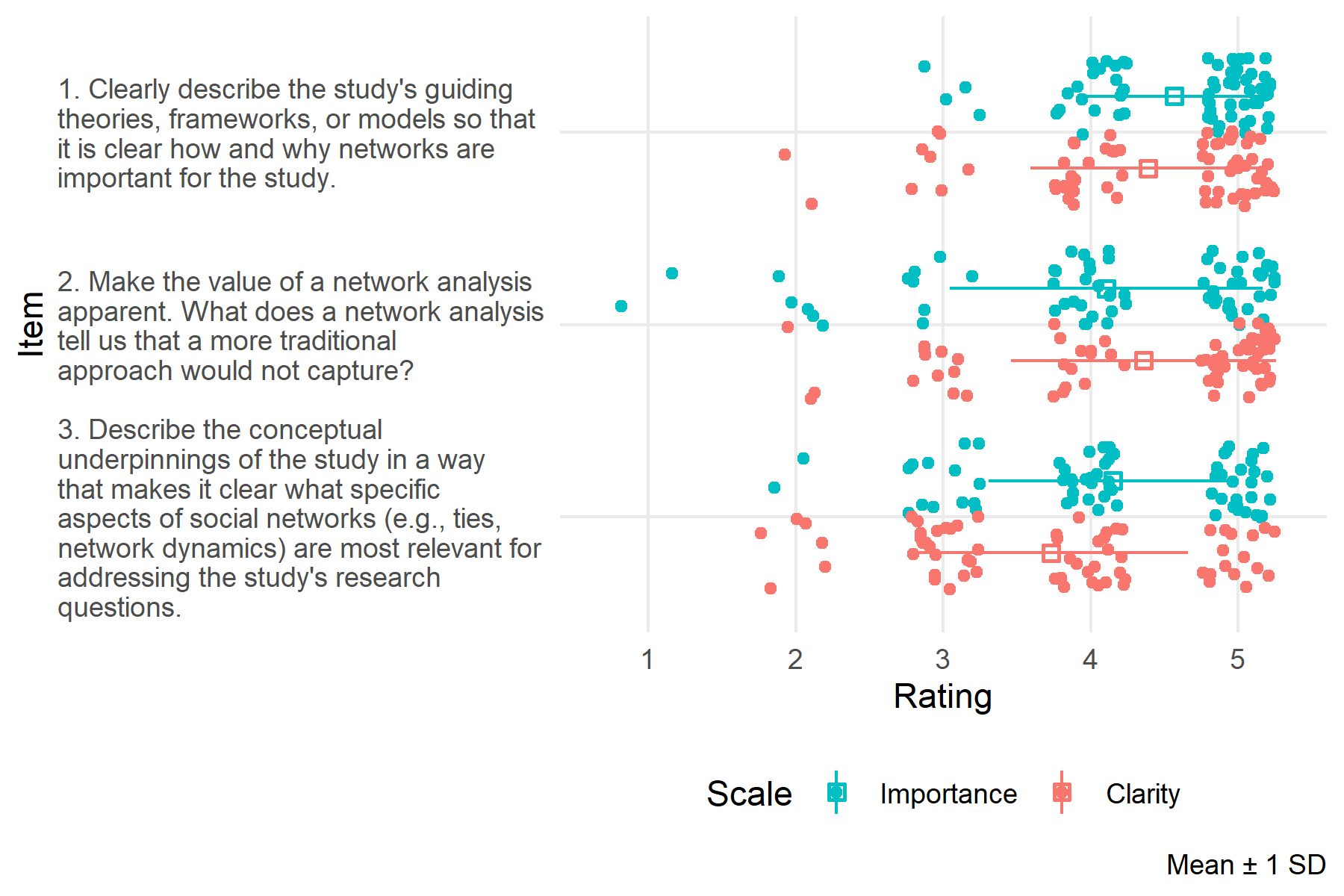
Conceptualization:

Operationalization:


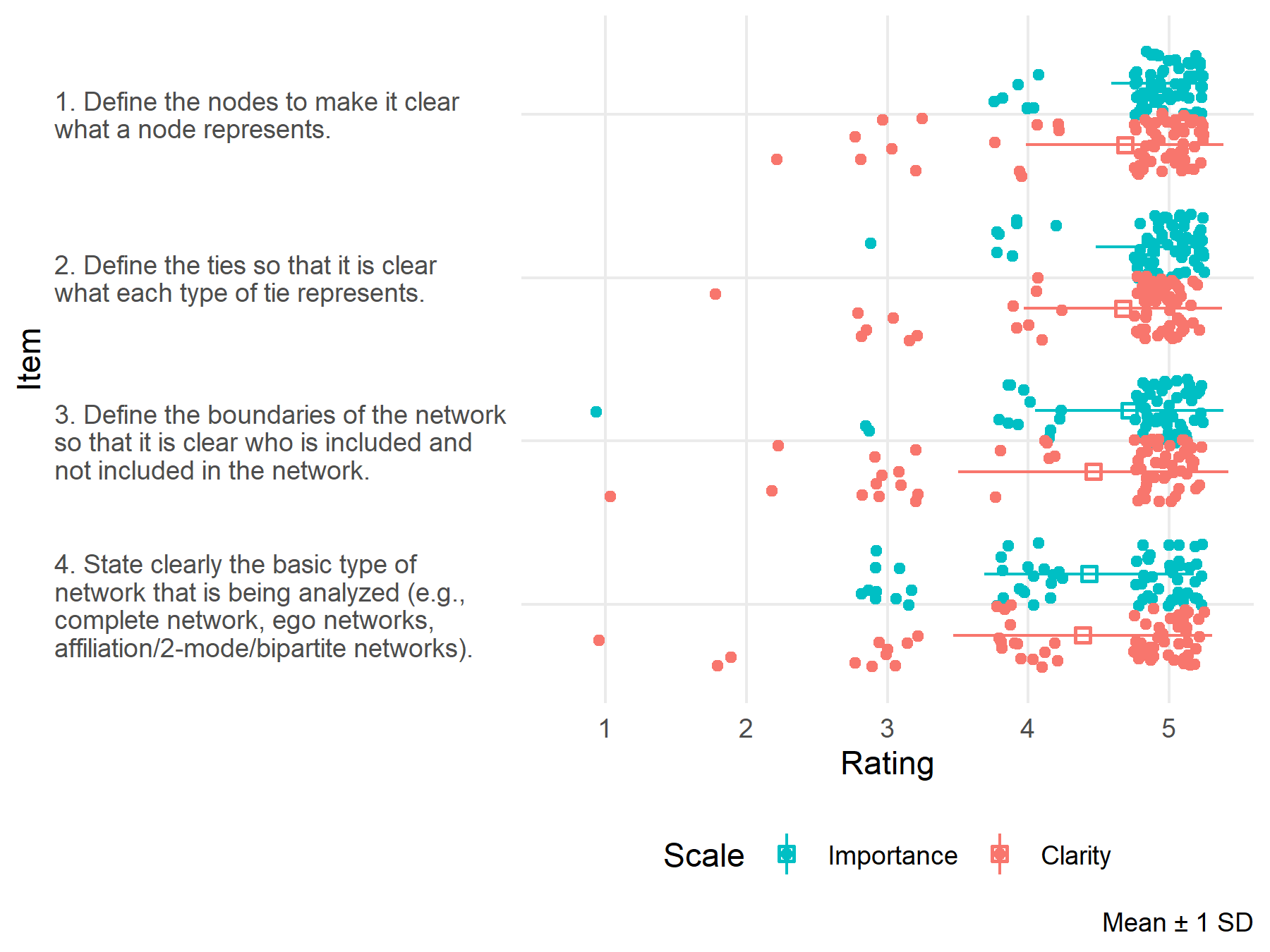


Data Collection & Management:


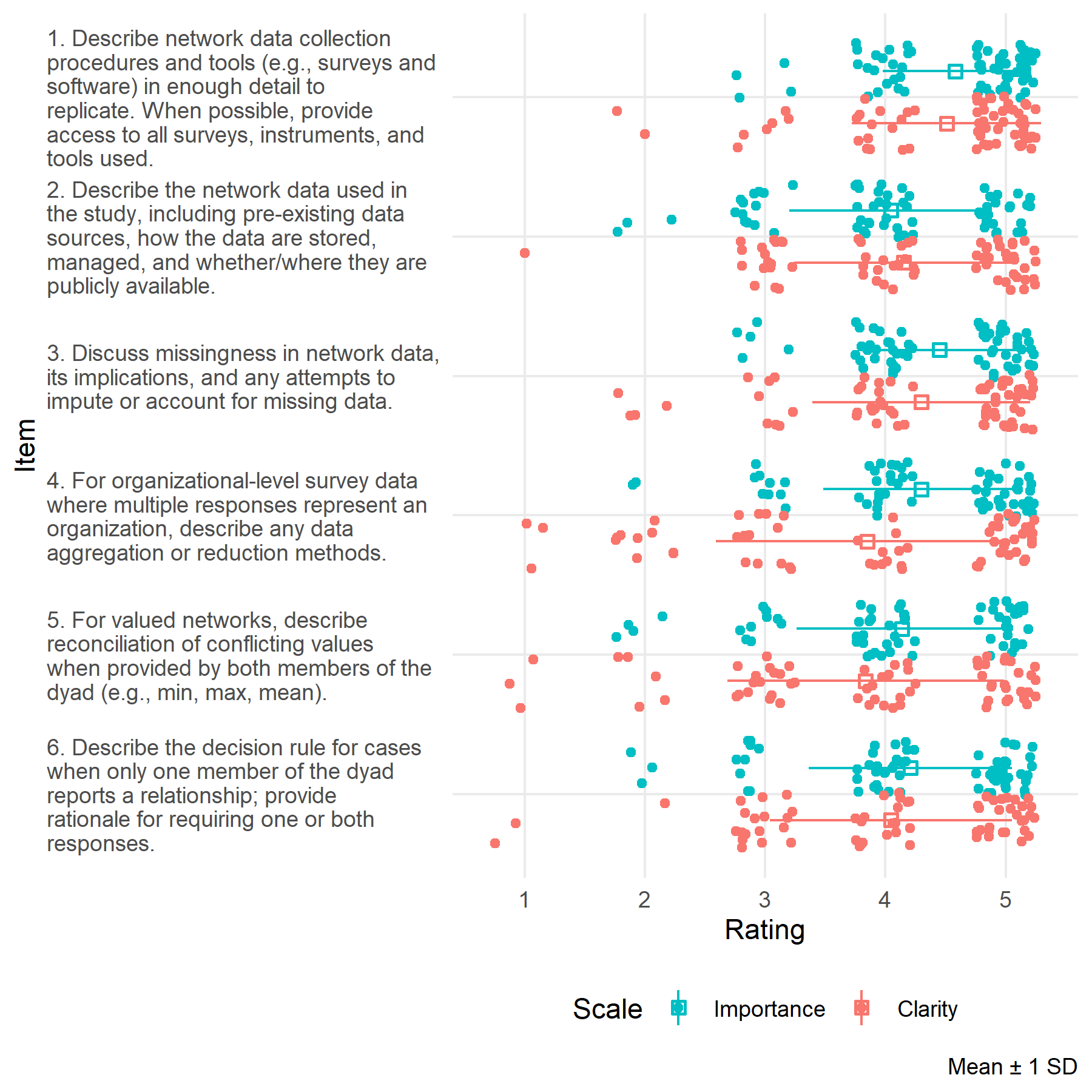


Analysis & Results Description:


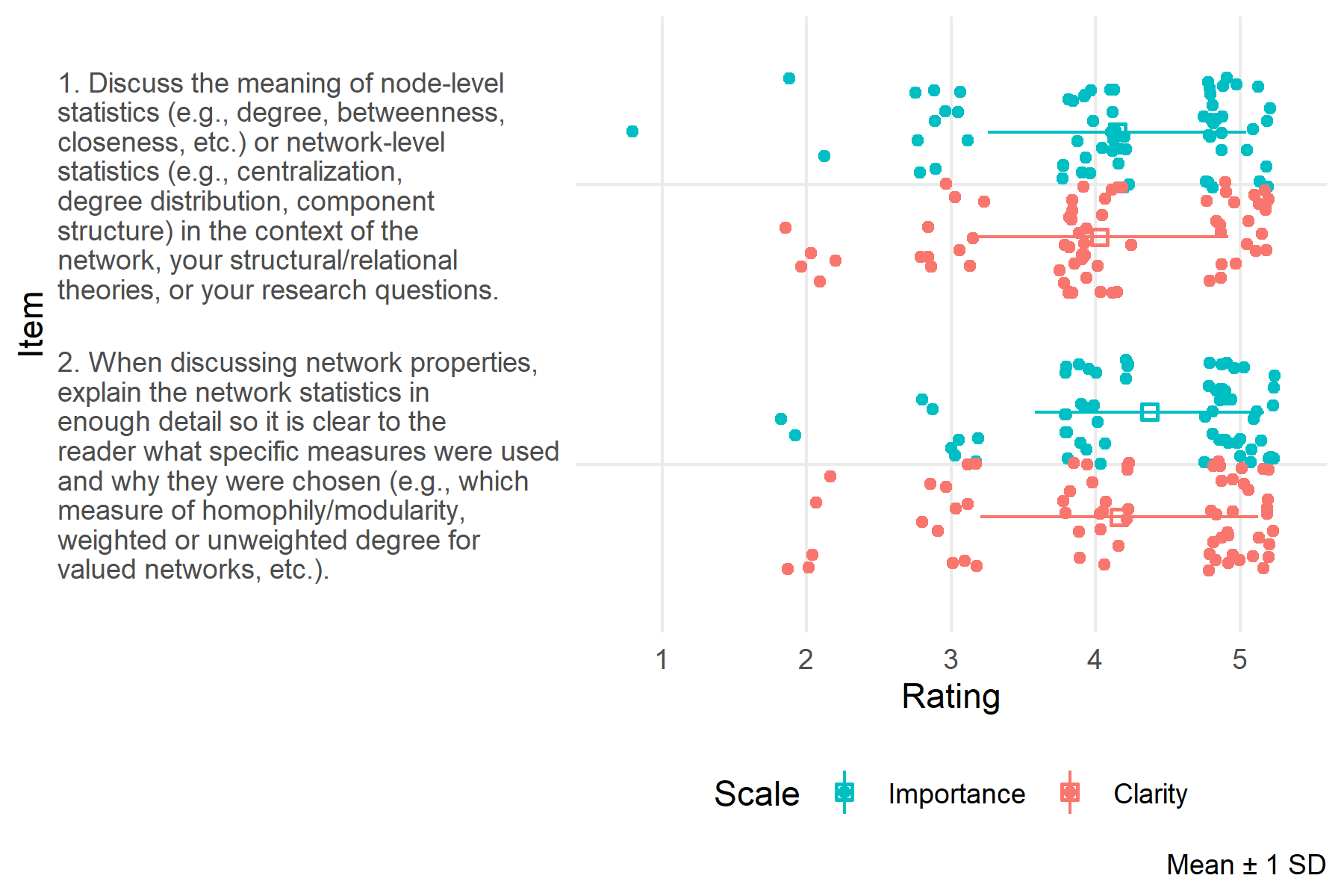


Analysis & Results Visualization:


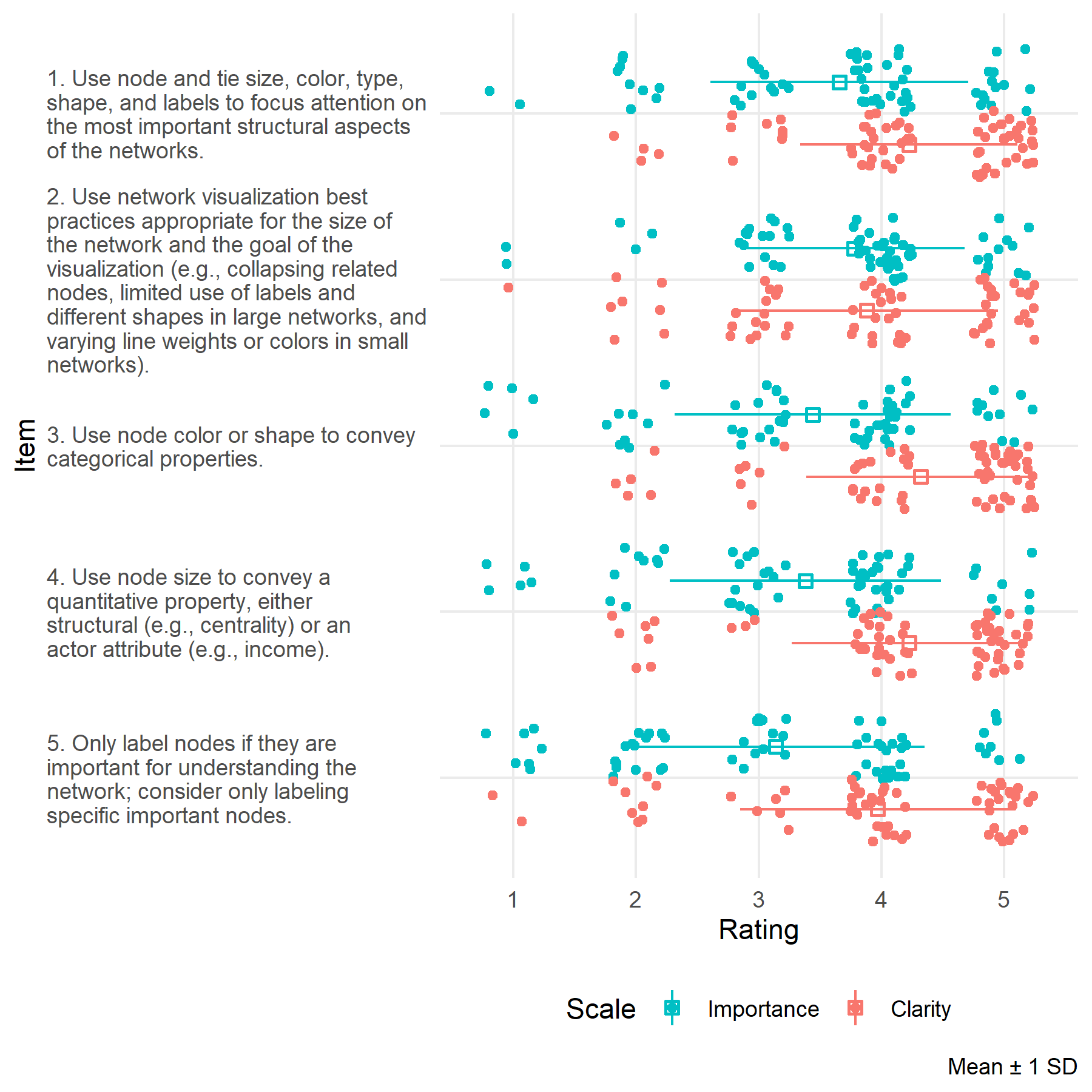


Analysis & Results Modeling & Simulation:


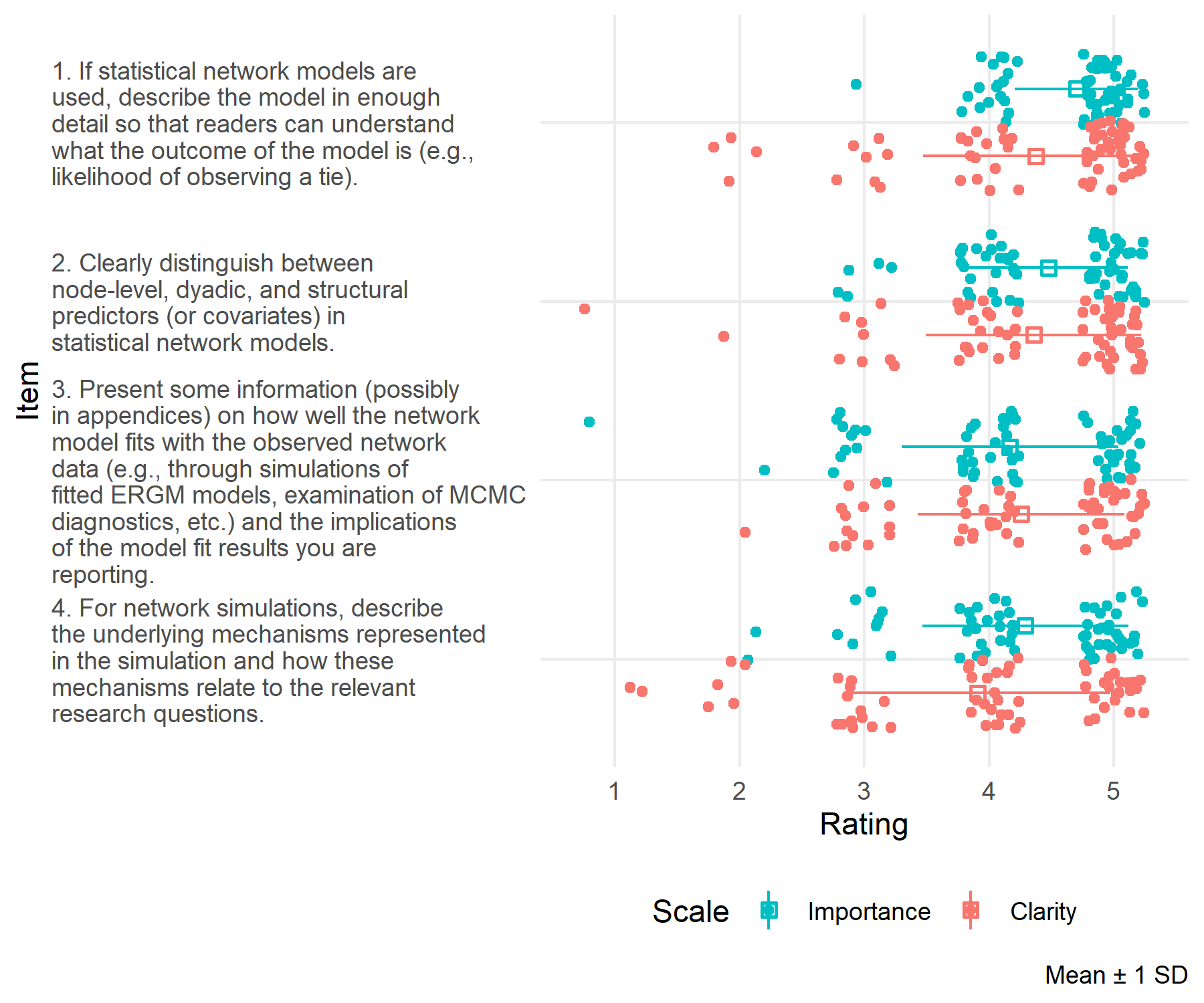


Ethics & Equity:


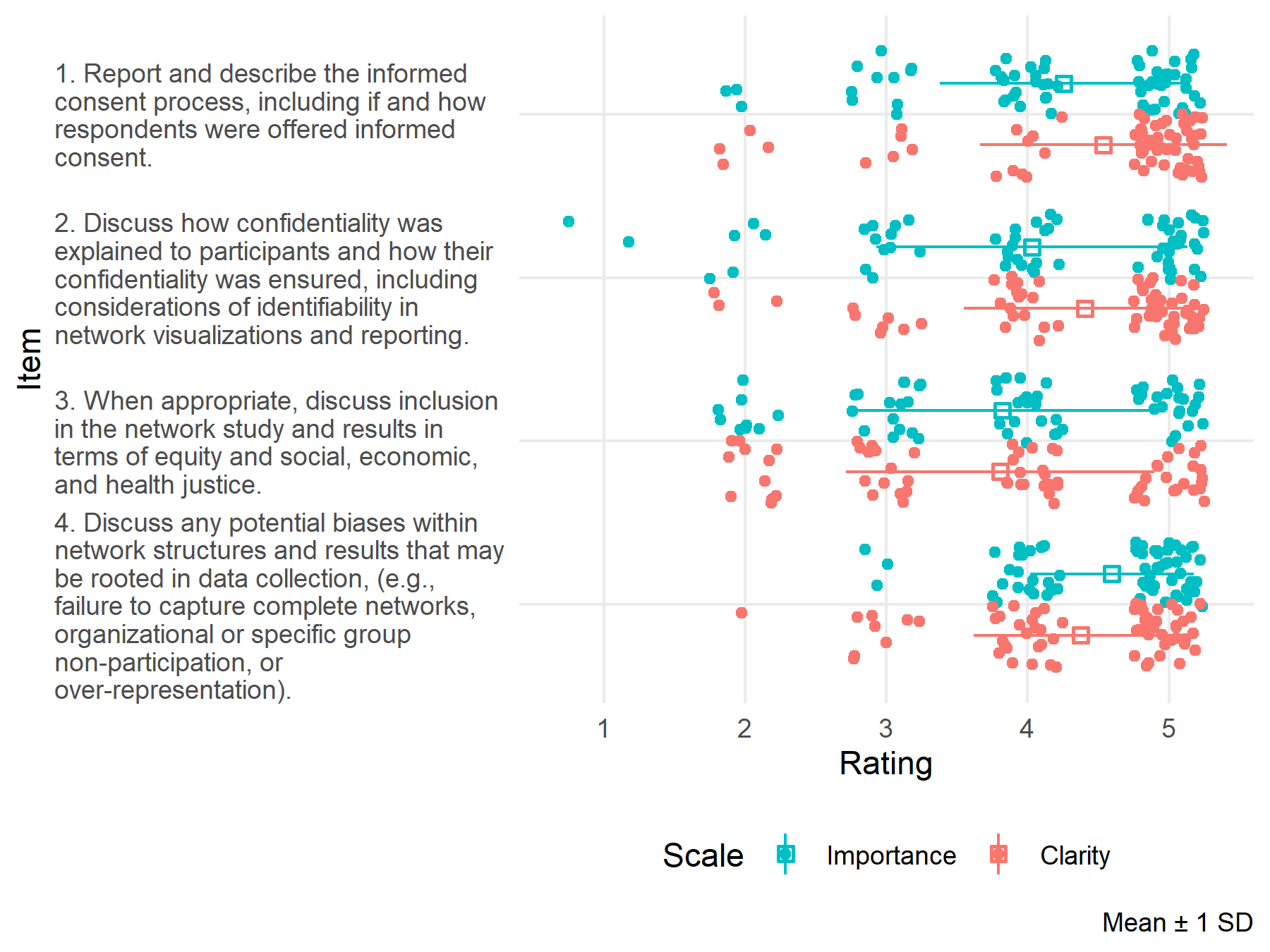

Supplement: S1 Fig — (DOCX) [file pone.0285236.s006.docx]
