# Supplementary material for: Introducing SoNHR–Reporting guidelines for Social Networks In Health Research
Source: PLoS One. 2023 Dec 14;18(12):e0285236. doi: 10.1371/journal.pone.0285236 (PMC10721040; doi:10.1371/journal.pone.0285236)
Supplement: Attachment — Submitted filename: Response to Reviewers.docx [file pone.0285236.s007.docx]

September 12, 2023

To PLOS ONE Editors and Reviewers,

We are pleased to submit our revised manuscript*, Introducing SoNHR – Reporting guidelines for social networks in health research.* We would like to thank our original reviewers and editors who have provided a wide range of useful critiques and suggestions. Our revised manuscript incorporates edits and new material throughout based on these reviewer suggestions.

We think this manuscript is much improved, particularly with a better justification of the need for network study reporting guidelines within the health sciences, and how these guidelines may benefit health science through improvements in validity, equity, and replicability. Second, more information is presented on recommendation development methods and results, which we hope will help both reviewers and readers better understand how these guidelines came to be.

The following table provides specific information on how we responded to each of the reviewers’ points, with links to where the new material can be found in the accompanying manuscript.

| *Critique* |  | *Response* |
| --- | --- | --- |
|  |  |  |
| R1 - *I think the motivation of this study could be strengthened and explained better. I appreciate that your idea is to draw a parallel between the benefits of reporting guidelines for other aspects of health science and the need for similar guidelines for network studies. Although I find this rationale meaningful, I would encourage you to contextualize your motivation more explicitly, explaining why reporting guidelines are needed for network science and what the risks/implications are of ignoring this need* |  | We have expanded the introduction to provide a stronger rationale for the guideline development in two different ways. First, by explicitly recognizing the role of reporting guidelines in addressing the current reproducibility crisis in the health and social sciences. Second, by adding a new paragraph that describes how network studies in the health sciences have their own distinctive validity, data, analytic and historical characteristics, supporting the general approach we took for developing the network reporting guidelines for the health sciences. (See lines 65-69, 79-81, 88-99, 107-110.) |
|  |  |  |
| R1 - *Furthermore, the category of "network studies in health sciences" is potentially very broad. Do you plan to develop a set of guidelines that suit any type of network studies/level of analysis/research questions? I think it would be worth clarifying this upfront as well as providing examples of what studies you have in mind and/or mentioning sub-streams of literature.* |  | The reviewer is correct that ‘network studies in health sciences’ can be broad, but this boundary is still useful with respect to intended audiences, usage, and domain focus. We are guided by Lisa Berkman’s conceptual framework that lays out how social networks are embedded within social-ecological systems driving human health, disease, and behavior (see lines 398-401).  The additions to the introduction described above should help clarify how these guidelines, while broad, are still intended for specific disciplinary applications. Also, the new section in the Discussion Section starting on p. 28 (lines 398-428) speaks to some specific health discipline applications such as disease transmission and psychological well-being.  In addition, we have added some additional references to how the guidelines will be disseminated, particularly by using the EQUATOR reporting guidelines for health research network and website. (107-110) |
|  |  |  |
| R1 - *I am not fully convinced by the approach you adopt to draft the initial 28 recommendations. While I do not doubt your extensive experience in the field (and the advantages of drawing on this), I believe your paper would benefit from the adoption of a more robust approach. This would also make the readers more confident about the conclusions you draw and ensure this initial list is not biased. This is relevant because – to the best of my understanding (pp. 10 and 12)– you asked the experts to provide suggestions on how to improve the listed recommendations, but not to indicate additional ones. You could conduct a systematic – instead of an informal – literature review and arrange the findings into a table, where you list the most recurrent recommendations (setting a reasonable threshold for “recurrent”) that you can derive from extant literature. Then, you could match them with your own experience – briefly explaining and justifying the recommendations you have derived from your own work.* |  | A systematic review would typically be a useful part of recommendations development, as the reviewer suggests. However, in this case we quickly realized it would not be possible. There are no other published studies focused on *reporting recommendations* (as opposed to network methods applications) that we found. Therefore, a systematic review is not possible here. We have added some additional clarifying information about this in the Methods section. (128-131) |
|  |  |  |
| R1 - *You report that the response rate to your expert panel survey is 45.6 %. I would encourage you to comment on this response rate, specifically on non-respondents. Did you check that the sample of respondents is not biased? Does it cover all key characteristics that you used to identify the initial sample?* |  | Thanks for this suggestion. We have edited and expanded the *Participants* section with new and clarifying information. (172-176).  We now are clearer on what our recruitment goals were, especially for ensuring an experienced and diverse participant group. (166-168)  We have added domestic/international participation numbers to Table 2. (190)  Also, despite limited information on our non-respondents, we were able to compare participants to non-respondents on institutional location and gender. (172-176). Taken together, this new information does support our interpretation that the respondents represent an experienced and diverse group of network and health scientists who were well-suited to inform the development of the network reporting guidelines. (183-186) |
|  |  |  |
| R1 - *It seems to me that most of the guidelines you provide apply to network studies across a variety of fields, not to health sciences specifically. Given the focus of your study, I encourage you to discuss in more detail – and in a more compelling way – the reasons why your guidelines are beneficial to health sciences, which drawbacks of existing studies they would allow addressing, which implications they would have for the reliability of results and policy implications. I appreciate your attempt to convey this message by explaining which audiences would benefit from your study, but I would recommend that you expand this section providing examples of research questions, empirical settings, etc. This consideration resonates with my comment on the introduction – and I think addressing both comments would strengthen your contribution.* |  | We have responded to these good suggestions in two ways. First, we have significantly expanded our rationale for health-science specific network guidelines. See our responses above, as well as new material in the introduction (especially 88-99). We have also edited Table 5 to make it clearer how these example studies come from the health sciences and are presented in ways that are consistent with the SoNHR guidelines. (starting on line 370).  Second, we have added a whole new subsection of the Discussion section called ‘Benefits to the health sciences’ where we directly engage with the suggestions here. This new section more explicitly presents how usage of these guidelines may benefit health science in three broad ways: enhancing validity, equity, and replicability. This section also connects more clearly to the material in Table 5, which we intended as the main way to illustrated specific health science research contexts for network studies. (398-429) |
|  |  |  |
| R2 - *The initial set of recommendations that the authors provide are based on an “informal review”. The authors need to clarify what this exactly means and provide concrete measures to showcase how this review was conducted. The authors need to clarify what “their experience using network methods is” and substantiating this claim to show how exactly this is relevant in the set of recommendations provided.* |  | We have added some additional information on what we mean by an informal review, specifically that we did rapid reviews of both network applications in the health sciences, as well as published papers focusing on reporting recommendations (see new material in the methods section, especially lines 128-131).  We have expanded on the author team’s expertise on network methods. (134-137). We did not feel comfortable referring to our own network scholarship in the manuscript, but collectively the author team has published many dozens of empirical network studies in the health sciences. This publication record should be available to the reviewer(s) since authorship is not blinded in PLOS ONE. |
|  |  |  |
| R2 - *More empirical analysis are needed in the first stage of the set of recommendation* |  | We have added more details in the methods and results sections on how the final guidelines were developed. In particular, we now present more information on how we selected our participant sample, and how the actual participants compared to the larger invitee list (166-176). In addition, we present more specific details about what kinds of responses we received in the qualitative response portion of the Delphi survey (154-156, 220-226) . We have also provided more detail on how the quantitative and qualitative results were used to form the final version of the recommendations (220-226, 237-240). |
|  |  |  |
| R2 - *Results are well presented. However, the set of 18 recommendations are quite generic at the moment. The recommendations can benefit from concrete examples specifically contextualised to the setting.* |  | We have made edits and added some new material to help ground the recommendations in specific research contexts. First, we have retitled Table 5 to make it clearer that this is a detailed list of actual health science studies that use network methods in ways that are consistent with the SoNHR recommendations (370). (As stated above, we always intended the studies presented in Table 5 to serve as examples of how these recommendations are tied to specific health science research questions and contexts.)  Second, we have added a new subsection of the Discussion section which provides some additional treatment of how the recommendations can benefit health science, particularly in terms of validity, equity, and replicability (398-429). |
|  |  |  |
| R3 - *Thanks for giving me the opportunity to read and review the paper. I found it interesting for the potential implications that are described, as it highlights the relevance of social sciences - and SNA more in particular - in health care studies. Unfortunately, I was not captured by any original contribution the paper should have produced. Recommendations are presented as very broad, not focused on the specific setting under investigation, and I did not find any original idea on how SNA methods may be adapted to health care. This is originally what I expected to find in the manuscript. Even if I appreciated very much the efforts of cross fertilization, again, I did not find an extact streamline the paper would contribute to.* |  | Hopefully, some of the new additions and edits we have made to the manuscript will have made it clearer how this work is an original contribution to the network and health science literature (see details above).  Also, we have hopefully made it clearer that this is not a paper that gives guidance for how SNA methods should be *used* in the health sciences, there are numerous texts and publications that already do this (see particularly lines 65-69, 102-107, 128-131, and 431-434). Rather, this paper presents new recommendations for how network methods and results should be *reported* when used by health researchers. |
|  |  |  |
|  |  |  |

Please let us know if you have any further questions, and thanks for your consideration.

Sincerely,

Douglas A. Luke, PhD

Director, Center for Public Health Systems Science

Irving Louis Horowitz Professor in Social Policy
