# Supplementary material for: Introducing SoNHR–Reporting guidelines for Social Networks In Health Research
Source: PLoS One. 2023 Dec 14;18(12):e0285236. doi: 10.1371/journal.pone.0285236 (PMC10721040; doi:10.1371/journal.pone.0285236)
Supplement: Attachment — Submitted filename: Response to Reviewers 2.docx [file pone.0285236.s008.docx]

November 17, 2023

To PLOS ONE Editors and Reviewers,

We are pleased to submit the 2^nd^ revised version of our manuscript*, Introducing SoNHR – Reporting guidelines for social networks in health research.* There were only a couple of minor concerns voiced by one of the reviewers, they have been addressed in this new version.

| *Critique* |  | *Response* |
| --- | --- | --- |
|  |  |  |
| R2 - Method section: the authors should better explain how they conduct "a rapid review", why this is justifiable (pg.7); and the use of only one database such as Scopus (figure 1). Also, they should clarify the keywords used to conduct this rapid review. |  | We have provided more details on the rapid review, see pages 7 & 8, lines 147 to 155. The rapid review was used to provide context and a jumping off point for the guideline development, but did not provide ‘data’ for the guidelines. That came primarily from the expert panel Delphi activities, as described in the paper.  We have added one new reference that helps support the rationale for using a rapid review.  Finally, we have added one new additional appendix that can be included in the supplemental materials. It provides more technical details of the Scopus review, including some explanation of why we chose Scopus (i.e., it’s the largest indexer of health science research in the world), and also includes the various keyword and search strings that we used, particularly for development of Figure 1. |
|  |  |  |

Please let us know if you have any further questions, and thanks for your consideration.

Sincerely,

Douglas A. Luke, PhD

Director, Center for Public Health Systems Science

Irving Louis Horowitz Professor in Social Policy
